# Unlocking the Role of Social Norms: How They Shape Women’s Public Toilet Usage in India

**DOI:** 10.4269/ajtmh.23-0220

**Published:** 2023-10-09

**Authors:** Karan Babbar, Upasak Das, Sania Ashraf, Alex Shpenev, Cristina Bicchieri

**Affiliations:** ^1^ Jindal Global Business School, O.P. Jindal Global University, Sonipat, India;; ^2^ Center for Social Norms and Behavioral Dynamics, University of Pennsylvania, Philadelphia, Pennsylvania;; ^3^ Global Development Institute, University of Manchester, Manchester, United Kingdom

## Abstract

Poor access to toilets has significant impacts on hygiene, health, safety, and well-being. Women in resource-poor areas may not use public toilets because of concerns about personal safety and the disapproval of others. This study examines social beliefs about women’s use of public toilets in India, using data from 5,052 households in rural, semi-urban, and urban slum areas of Bihar and Tamil Nadu in 2018. We asked respondents about their beliefs regarding the prevalence of young women aged 16 to 30 years using public toilets alone and whether this behavior was approved of in their community. We also asked about their personal beliefs on this issue. We used hypothetical vignettes to assess perceptions of a young woman’s behavior in different settings regarding public toilet usage by women. Our results show that people who believe many women in their community use public toilets alone and approve of it are more likely to have positive beliefs about this behavior. The experimental vignettes suggest a potential causal link between the prevalence and approval of public toilet usage among young women and their likelihood of using it. These findings are consistent across Bihar and Tamil Nadu and the three administrative regions, indicating that interventions aimed at changing social expectations about women’s use of public toilets should focus on highlighting community members’ usage and approval. Efforts to encourage woman’s access to public toilets and services should target shifting beliefs about public toilet usage among women without disapproval from others.

## INTRODUCTION

Sanitation is a human right that entitles everyone “*to have physical and affordable access to sanitation, in all spheres of life, that is safe, hygienic, secure, and socially and culturally acceptable, and that provides privacy and ensures dignity.*”^1^ Access to sanitation facilities with a special mention to attend to the needs of girls and women is recognized as one of the Sustainable Development Goals to be achieved by 2030. Improved sanitation leads to better public health, hygiene, and greater well-being and provides privacy and security to users.^2^ In India, efforts from recent national sanitation programs have increased access to toilets.^3^ However, according to a National Family Health Survey report, 19% of people in India do not have access to toilets, implying that the prevalence of open defecation is substantial.^4^

In resource-poor settings such as India, public toilets, which are managed communally or through the local administrative body, aim to provide access to adequate sanitation within the community. However, known structural and societal barriers lead to poor usage of public facilities in India, where many find them inaccessible, dirty, and unhygienic.^5^^–^^7^ Public toilets disproportionately impact women, as the unhygienic conditions can lead to higher levels of reproductive and urinary tract infections among females.^8^^–^^11^ In addition, community toilets are inadequate in number and not adequately managed or designed to capture the unique needs of women. A survey conducted by ActionAid India found no separate section for women in Delhi in 35% of the 229 surveyed public toilets.^12^ They also found that 45% of the toilets had no mechanism to lock the door from inside, and 53% of women’s toilets did not have running water.

Notably, existing gender norms governing women’s mobility are also adverse for women.^13^^,^^14^ Because of concerns about their safety combined with prevalent gender-based restrictions, use of public facilities may be considered unnecessary and unwarranted for women within the community. They face a higher risk of sexual and physical violence when using poorly lit or badly located public toilets.^15^ Previous studies have reported about queues of men outside public toilets teasing and abusing the women using the public toilets.^16^^,^^17^ A study conducted in two large cities in India (Pune and Mumbai) found a higher incidence of violence against women if they used public toilets alone at night.^18^ An exploratory study in Bhopal and New Delhi found that when using public toilets alone, women have faced insulting comments, brick throwing, stabbing, and even sexual assault.^19^^,^^20^ Extreme incidences were documented in Bihar, where nearly half of the reported rape cases in the state were “sanitation related”.^21^ Therefore, despite potentially higher marginal benefits of toilet usage for women, use of public sanitation facilities may be disproportionately lower among females.

To encourage public toilet usage among women, it is critical to understand how those in one’s community perceive women using public toilets alone and whether they approve of it. Among many factors, including individual-level ones such as education or age, are social beliefs and expectations surrounding public toilet usage among women. Does one’s perception about the prevalence of public toilet usage among women alone within the community influence one’s own beliefs? Does one’s perception of others’ approval of whether a young girl should use a public toilet alone matter? This paper answers these questions by utilizing social norms theory (SNT) to understand the link between social beliefs and individually held beliefs around public toilet usage by women. We examined these links using primary household survey data in urban slums and semi-urban and rural areas within Bihar and Tamil Nadu, India. This study aimed to extend these insights to recommend potential avenues for behavioral change interventions to leverage social beliefs and encourage public toilet usage among females.

### Social norms framework.

**Figure 1.**
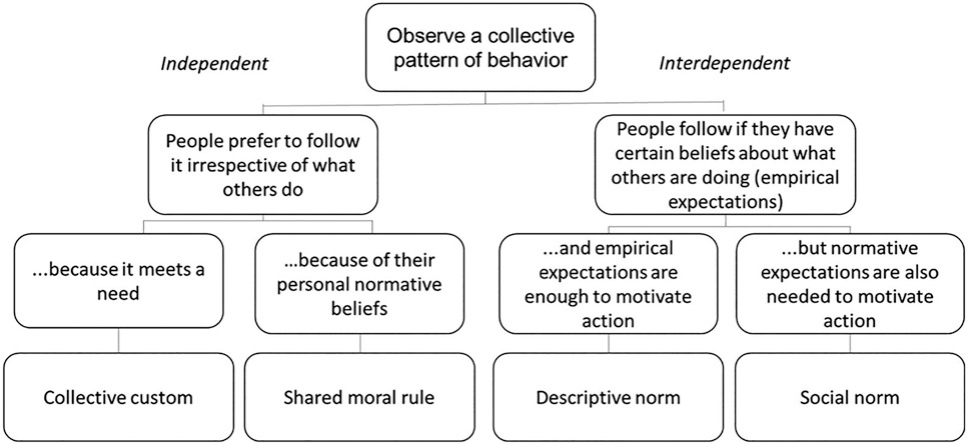
Categories of behavior under the social norms theory (SNT). Source: Bicchieri 2017.^24^

We used elements of SNT as propounded by Bicchieri^22^ to analyze and evaluate group behavior related to public toilet usage by women alone (Figure 1). The underlying research question was to assess whether the beliefs and behaviors of other members of one’s community or network guide individual beliefs and behavior. More formally, collective behavior is classified as independent if beliefs about what others do or approve of do not modify an individual’s behavior. Interdependent behavior, on the other hand, depends on what others in the close network or community do or approve of. In particular, interdependent behavior is motivated by social expectations, comprising two key elements: empirical expectations (EEs) and normative expectations (NEs). Empirical expectations refer to one’s perception about what others in the community/network/neighborhood do with regard to the behavior we are studying. On the other hand, NE refers to one’s perception of whether others in the community/network/neighborhood approve of that behavior. If the behavior is driven only by EE, the theory suggests that the behavior follows descriptive norms. However, if NE is also influential, social norms are understood to be motivating the behavior. It is important to note that personal normative beliefs (PNBs), which are personal beliefs about approval of a particular behavior, also play a key role in shaping behavior.

In this paper, we first examine the PNBs around the usage of public toilets by a woman alone and whether they can be predicted by the existing social expectations (EEs and NEs). We then use experimentally manipulated hypothetical vignettes to understand how random variation in these expectations can potentially influence behavior. In the process, the study allowed us to think of interventions that can be used to encourage greater public toilet usage, especially among women.

## MATERIALS AND METHODS

### Data collection.

A cross-sectional survey was conducted from April to June 2018 in Bihar and Tamil Nadu. Each state was broadly categorized into three regions based on sociocultural characteristics and sampled across three types of settlements: rural, semi-urban, and notified slums in urban areas. Individuals living outside these areas were not included in the sampling strategy. Further details are included here.^23^

For the rural sample, one community development block was randomly selected from the list of blocks in each selected district. From this block, one gram panchayat (GP) was randomly chosen, and another GP was then selected as a match based on its socioeconomic characteristics, which included population size, proportion of individuals in socially disadvantaged social groups (scheduled caste and scheduled tribe), illiterate individuals, total laborers, and households with latrines based on the 2011 census. Similarly, for the semi-urban sample, two town panchayats (TPs) were chosen from that same district (TPs in Tamil Nadu and nagar panchayats in Bihar). From the selected towns, three census wards were chosen at random and surveyed. For the sample of urban slums, we randomly selected one municipal corporation (MC) from each of the sociocultural regions (SCRs). An SCR was formed by grouping districts that shared linguistic homogeneity, geographical contiguity, financial and economic similarities, administrative uniformity, and a regionalization of culture and lifestyle.^25^ From the chosen MCs, two notified slums, defined as all notified areas in a town or city notified as “slums” by a government authority, were selected randomly. This survey does not include individuals living in non-notified slums; therefore, this non-inclusion should be considered a limitation of our study.

Before respondents were chosen, a thorough listing of all residing units and households in the areas selected for the survey was conducted. The listing operation entails traveling to each of the chosen primary sampling units (PSUs), sketching a map of the buildings there, and then noting the names of the household heads and the ages of all eligible people living in each of the households within the PSUs.

A census for the entire age-eligible population was conducted for each sampling unit, and a random sample was chosen for the survey. Our sample was not representative at the state level; however, it was large enough to understand variations in the beliefs and norms within the geography type. The study sites received different levels of toilet construction interventions from various national, state, and non-governmental organization (NGO) interventions.

To enhance the validity of our survey, a team of bilingual researchers translated the survey into the local language of Hindi/Tamil and then back-translated it into English. To ensure standardized data collection, we conducted a 10-day survey training with the team and another 3-day training of the local survey trainers across the states. The surveyors used handheld tablets to collect data. Field workers secured verbal informed consent before administering the survey. The data were gathered using computer-assisted personal interviewing on portable tablets. University of Pennsylvania researchers used deidentified data to conduct the analysis.

### Measures.

We collected data on various individual and household characteristics and perceptions of social norms and community sanctions regarding public toilet usage of women alone. A series of survey items were used to understand individuals’ beliefs about public toilet usage by women alone, in line with the social norm’s framework. The framing of survey items was informed by formative research, which included focused group discussions with young women. Specific to social beliefs and expectations, the following questions were asked during the survey.

1. PNBs: “Society may think it is right or wrong for a woman between the ages of 16 and 30 to leave the home alone to use the public toilet. Do you personally think it is right, neither right nor wrong, or wrong for a woman between the ages of 16 and 30 to leave the home alone to use the public toilet?”
2. EEs: “Out of 10 women between the ages of 16 and 30 in your community, the last time they left the house to use the public toilet, how many do you think did so alone?”
3. NEs^†^: “Out of 10 members in your community, how many do you think believe that it is wrong for a woman between the ages of 16 and 30 to leave the home alone to use the public toilet?”

Please note that the question on EEs was asked only of respondents who reported having a community or public toilet within a 15-minute walking distance from their place of residence. Therefore, out of the total number of respondents, only 1,345 individuals provided responses to this particular question. However, the question on NEs is relevant to all respondents, and thus it was asked of all 5,052 individuals.

We used randomly assigned hypothetical vignettes to evaluate the conditionality of public toilet usage by women alone and to comprehend the potential impact of EEs and NEs. Here, we experimentally manipulated the level of EEs and NEs around young women going outside to use a public toilet alone and assessed its impact on the expected behavior of a fictional character in a short story. In particular, each respondent was given a random vignette with a combination of high or low EEs and high or low NEs, and the respondent was then asked what the vignette character might do. This allowed us to measure the degree to which a change in EEs or NEs leads to changes in a respondent’s assessed likelihood that the hypothetical character would use the public toilet alone. Because the vignettes were randomly manipulated, we were able to draw a potentially causal link connecting social expectations and behavior. We were able to assess changes in behavior against four possible randomly assigned vignettes: low EE and low NE, high EE and low NE, low EE and high NE, and high EE and high NE. In our survey, the question in the following box was asked:

Please imagine an area similar to where you live.

A young woman from your area, whom you don’t know, moved there 1 year ago.

She uses a public toilet when she needs to defecate.

She learned that [most]/[few] people disapprove of women going to the public toilet alone, [and]/[but] she also learned that [most/few] women go alone.

What do you think she will do?

In the above vignette, the phrase “*most/few*” is randomly changed to arrive at one of the four combinations. The survey items were translated into Hindi and Tamil with the help of a local Hindi and Tamil language expert. A detailed discussion was done with the Hindi and Tamil language experts to ensure that the translation did not change the meaning of the survey items. These items were then back-translated into English by another independent translator. Feedback from the respondents received during the pilot test was incorporated into the final set of items before they were administered to the study sample.

### Empirical strategy.

To estimate the effect of public toilet usage by women alone (EEs and NEs on the PNBs), we first used a simple probit regression, which uses the maximum likelihood estimation to yield the estimates. We used the following regression equation:

$$\mathrm{Pr}\left(Y_{\text{ihp}}=1\right)=F\;({\text{β}}_{0}+{\text{β}}_{1\;}{\text{EE}}_{\text{ihp}}+{\text{β}}_{2\;}{\text{NE}}_{\text{ihp}}+{\text{β}}_{3\;}X_{hp}+{\text{β}}_{4\;}I_{\text{ihp}}+{\text{β}}_{5\;}{\text{π}}_{p})$$

Here, $Y_{\text{ihp}}$ is the outcome variable, which takes the value of 1 if the respondent, i, from a household, H, situated in a PSU, p, feels it is right for a woman to leave home alone to use the public toilet and 0 otherwise. ${\text{EE}}_{\text{ihp}}$ denotes the associated EE, a continuous variable that reports the number of women (out of 10) between the ages of 16 and 30 years in their community who leave the house alone to use the public toilet. ${\text{NE}}_{\text{ihp}}$ denotes the associated NE, a continuous variable that reports the respondent’s beliefs about how many in the community feel it is right for women to leave home alone to use the public toilet. In the equation, $X_{hp}$ denotes the sociodemographic and household variables including gender, social group, religion, education level, assets, toilet ownership, and household location (rural, urban, semi-urban) dummies. The individual-level covariates were accounted for through the vector, $I_{\text{ihp}}.$ We further controlled PSU fixed effects through ${\text{π}}_{p}$, which captured the time-invariant PSU-level characteristics. ${\text{β}}_{1}$ and ${\text{β}}_{2}$ are the estimated marginal effects of EEs and NEs, respectively. All the standard errors were clustered at the PSU levels.

Past literature on social norms has pointed out that people tend to perceive their behavior as the most common perception in the community^26^^,^^27^ Using hypothetical vignettes, we manipulated the levels of EE and NE via a story and measured their impact on the person’s behavior as anticipated by the respondent. As explained, these vignettes were controlled using a 2 × 2 plan. Every respondent was presented with a randomly selected single vignette with variations across the lower and higher levels of EEs and NEs. Using this, we were able to measure changes in the anticipated conduct of respondents based on changing social expectations. We regressed the following equation to assess the potential influence of social belief on anticipated hypothetical character behavior:

Pr(Yihp=1)=F(β0+β1(lowEEihp lowNEihp)      +β2 (highEEihp lowNEihp)      +β3(lowEEihp highNEihp)      +β4(highEEihp highNEihp)      +β5 Xhp+β6 Iihp+β7 πp)

 In the above equation, ${\text{lowEE}}_{\text{ihp}}{\text{lowNE}}_{\text{ihp}}$ denotes the vignette with low EEs and low NEs. Similarly, ${\text{highEE}}_{\text{ihp}}{\text{lowNE}}_{\text{ihp}}$ denotes the vignette with the high EEs and low NEs. The others follow the same rule. Because these vignettes were randomly assigned to the respondents, we argue that the responses are potentially linked causally to the conditions given in the vignette. Our estimates (i.e., ${\text{β}}_{1}$ to ${\text{β}}_{4}$) refer to the impact of the varying levels of EEs and NEs on the respondent’s predicted behavior. $X_{hp}$ denotes the sociodemographic and household variables including gender, social group, education levels, assets, and toilet ownership. We also controlled the PSU type and PSU fixed effects.

Previous studies have used laboratory experiments to understand how social beliefs (EEs and NEs) affect PNBs for certain behaviors.^24^ In these settings, hypothetical vignettes provide an alternative way to assess the impact of social beliefs on behavior. The fact that we told stories about a fictitious character (a young, newly relocated woman) and placed her in hypothetical situations in these randomized vignettes is noteworthy.^28^^,^^29^ Previous studies have argued that asking people about these hypothetical situations can induce their true beliefs and expectations, especially when the questions are socially sensitive and the responses could be influenced by social desirability biases.^28^^,^^30^ These vignettes change the hypothetical character’s social circumstances (instead of the participant’s) before asking about the character’s likely behavior, so they can offer a nonthreatening way to explore expectations, beliefs, or attitudes on an issue of concern.^24^ In addition, we contend that any inherent bias would be corrected in the regressions as we controlled for sociodemographic and household variables in addition to EEs and NEs. The existing literature has used similar experimentally manipulated and randomly assigned vignettes to study exclusive breastfeeding and toilet usage behaviors.^31^^,^^32^

## RESULTS

### Characteristics of study participants.

A total of 5,052 respondents participated in our study. Table 1 presents the basic descriptive statistics of the socioeconomic and demographic characteristics of these respondents. They also represent the set of covariates we used in the regression. Supplemental Table 1 provides a detailed definition of each of these variables. The mean age of the respondents was 37 years, and there was an equal split between men and women across Bihar and Tamil Nadu. Hinduism was the primary religion across both states. Almost one-quarter of our respondents in Bihar were Muslims, representing only 8% of the population in Tamil Nadu. Respondents belonging to the other religions, primarily Christians (∼7%), were found in Tamil Nadu and represented around 1% of the sample in Bihar. These differences in religious composition are in line with the data from these two states.

**Table 1:** Sociodemographic characteristics of surveyed respondents, Bihar and Tamil Nadu, 2018^33^

Characteristic	Overall	Bihar	Tamil Nadu	Urban	Semi-urban	Rural
Gender						
Male	50.16	48.12	52.20	49.82	49.97	50.69
Female	49.84	51.88	47.8	50.18	50.03	49.31
Religion						
Hindu	79.37	73.71	85.07	80.24	78.24	79.70
Muslim	16.65	25.42	7.82	17.77	12.72	19.64
Other	3.98	0.87	7.11	01.99	09.04	0.66
Social group						
SC/ST	34.19	28.79	39.26	45.35	28.30	29.03
Other	22.94	14.26	30.92	16.47	23.22	29.16
OBC	42.87	56.75	29.82	38.19	48.48	41.80
Owns a toilet						
No	37.17	41.77	32.55	31.27	32.99	47.49
Yes	62.83	58.23	67.45	68.73	67.01	52.51
Education						
No education	31.94	42.64	21.2	28.51	27.45	40.08
Primary	12.62	11.23	14.01	13.56	12.63	11.67
Secondary	31.78	27.14	36.44	32.85	33.33	29.08
Higher	23.66	18.99	28.34	25.08	26.59	19.17
Assets (in numbers)						
0	26.96	52.61	1.19	22.06	25.39	33.53
1	31.7	27.88	35.33	34.72	31.72	28.64
2	24.71	12.72	36.76	23.81	26.02	24.23
3	16.63	6.79	26.52	19.41	16.87	13.60
Total sample	5,052	2,533	2,519	1,660	1,737	1,655

OBC = other backward caste; SC/ST = scheduled caste/scheduled tribe. The numbers presented in the table are percentages. The study uses the LENNS Survey data from Bihar and Tamil Nadu, 2018.^33^

Two-fifths (∼41%) of our respondents belonged to the other backward caste category, whereas 34% belonged to the scheduled caste (SC)/scheduled tribe (ST) category. Other backward castes represent a higher proportion of respondents (∼52%) in Bihar, and those of SC/ST (∼38%) were more commonly found in Tamil Nadu, which is in line with the social class composition of these states. As one would expect, more people in Tamil Nadu (∼28%) had completed higher education than those in Bihar (∼19%). Around 63% of the respondents owned a private toilet, with 58% from Bihar and 67% from Tamil Nadu (Table 2).

**Table 2:** Empirical and normative expectations across states and gender

Expectation	India	Bihar	Tamil Nadu	Male	Female
EE	5.31 (3.10)	4.02 (3.69)	5.74 (2.74)	4.87 (2.98)	5.71 (3.16)
NE	5.45 (3.44)	4.76 (3.53)	6.13 (3.20)	5.27 (3.26)	5.62 (3.60)

EE = empirical expectation; NE = normative expectation. The mean is presented in the table along with the SD in parenthesis.

Regarding EEs, about 13% of the people believe that none of the women in their community went alone to use the public toilets, and about 56% believe that at least half of them did. About 14% reported that all women in their community went to public toilets alone for defecation. Regarding NEs, about 13% of the women believe none in their respective community approve of women going to the public toilet alone, whereas 24% believe that all community members approve of this behavior.

We found that more women (57%) than men (49%) believed that women in their community go alone to use the public toilets. However, only slightly more women (56%) than men (53%) said they approve of women using public toilets alone.

### Association of social beliefs with PNBs held by individuals.

Table 3 gives estimated results from the regression outlined in Equation (1). We present six specifications: The first and second specifications have the indicators of EE and NE individually in the model without any controls; the third specification has the indicators of EE and NE together; and the fourth specification has the indicators of EE and NE together along with the controls. The fifth and sixth specifications are similar to the fourth specification; however, they are restricted to the data in Bihar and Tamil Nadu, respectively. The last three specifications have the most comprehensive set of controls, which allowed us to get close to unbiased estimates and include district, state,^‡^ PSU type, and PSU name fixed effects. We assessed whether social expectations (empirical and normative beliefs) were associated with individual beliefs (PNBs), which indicates whether they personally think the behavior was the right thing to do (PNBs).

**Table 3:** Marginal effects to estimate the association of empirical and normative expectations with the personal normative beliefs of public toilet usage by women alone

Variable	It is right for women to use public toilets alone (PNB)					
	Full sample				Bihar	Tamil Nadu
	(1)	(2)	(3)	(4)	(5)	(6)
Leaving house alone to use public toilets (EE)	0.05*	–	0.03*	0.03*	0.02*	0.04*
	(0.003)	–	(0.003)	(0.005)	(0.007)	(0.004)
Approval of leaving the house alone to use public toilets (NE)	–	0.07*	0.06*	0.06*	0.06*	0.06*
	–	(0.001)	(0.002)	(0.003)	(0.005)	(0.003)
Gender						
Male	–	–	–	–	–	–
Female	–	–	–	0.06	−0.05	0.11*
	–	–	–	(0.039)	(0.047)	(0.026)
Social group						
SC/ST	–	–	–	–	–	–
Other	–	–	–	0.10†	−0.01	0.13‡
	–	–	–	(0.058)	(0.102)	(0.038)
OBC	–	–	–	0.04	−0.02	0.07†
	–	–	–	(0.043)	(0.057)	(0.034)
Owns a toilet						
No	–	–	–	–	–	–
Yes	–	–	–	−0.02	−0.01	−0.03
	–	–	–	(0.031)	(0.053)	(0.028)
Education						
No education	–	–	–	–	–	–
Primary	–	–	–	0.02	0.14*	−0.04
	–	–	–	(0.043)	(0.073)	(0.041)
Secondary	–	–	–	−0.02	−0.08	−0.03
	–	–	–	(0.028)	(0.068)	(0.035)
Higher	–	–	–	0.02	0.14‡	−0.05
	–	–	–	(0.043)	(0.074)	(0.038)
Assets						
0	–	–	–	–	–	–
1	–	–	–	−0.01	−0.03	0.09
	–	–	–	(0.040)	(0.063)	(0.067)
2	–	–	–	−0.03	−0.13†	0.09
	–	–	–	(0.040)	(0.075)	(0.069)
3	–	–	–	−0.03	−0.14	0.10
	–	–	–	(0.042)	(0.083)	(0.071)
District FE	N	N	N	Y	Y	Y
State FE	N	N	N	Y	Y	Y
PSU type FE	N	N	N	Y	Y	Y
PSU name FE	N	N	N	Y	Y	Y
Pseudo R2	0.09	0.25	0.27	0.36	0.34	0.4
Observations	1,345	5,052	1,345	1,258	308	950

EE = empirical expectation; FE = fixed effects; N = no; NE = normative expectation; OBC = other backward class; PNB = personal normative belief; PSU = primary sampling unit; SC/ST = scheduled caste/scheduled tribe; Y = yes. The dependent variable is whether it is right or wrong for a woman between the ages of 16 and 30 years to leave the home alone to use the public toilet. Table 4 gives the marginal effects from the probit regression outlined in Equation (1), which reports the results of social beliefs (EEs and NEs) on PNB. We present six specifications: The first and second specifications have the indicators of EE and NE individually in the model without any controls; the third specification has the indicators of EE and NE together; and the fourth specification has the indicators of EE and NE together along with the controls. The fifth and sixth specifications are similar to the fourth specification; however, they are restricted to data in Bihar and Tamil Nadu, respectively. The last three specifications have the most comprehensive set of controls, which allowed us to get close to unbiased estimates and include district, state, PSU type, and PSU fixed effects. All standard errors were clustered at PSU levels.

* *P* < 0.01.

† *P* < 0.05.

‡ *P* < 0.1 level.

The probability of holding a positive personal belief that it is right for women to leave home to use public toilets alone increased as the perceived prevalence of women going out to use public toilets alone (EE) increased by about 3%. We documented an increase of 6% if they perceived others approved of the behavior (NE) (Table 3, column 4). Therefore, the likelihood of holding positive beliefs in the context of public toilet usage among females alone increases when its perceived prevalence and approval within the community increases.

We found qualitatively similar results when the sample of respondents from Bihar and Tamil Nadu were analyzed separately. Nevertheless, the association of EEs with PNBs regarding women using public toilets alone was lower among those from Bihar than among those from Tamil Nadu. However, in both states, the association remained statistically significant (Table 3, columns 5 and 6). When the urban, semi-urban, and rural areas were compared, we also found similar results, with the only exception of EEs not being statistically significant in rural areas§ (Supplemental Table 2).^‖^

### Estimation using randomly assigned vignettes.

Our analysis shows how respondents take cues from the community and that the community’s social beliefs affect their PNBs. Despite controlling for the various sociodemographic variables, respondents who felt it was right for women to go outside to use public toilets alone may be more likely to believe that more women in the community also use them alone. In other words, the respondents may form beliefs about public toilet usage by women alone within their community based on their own choice. Thus, to overcome this potential bias, we randomly assigned the vignettes to the respondents, each with four possible combinations of EEs and NEs: low EE–low NE, high EE–low NE, low EE–high NE, and high EE–high NE, as explained earlier. Because each of these combinations was randomly administered to the respondents, the exogenous variations in the type of vignette assigned ensured a marginal effect associated with these combinations, further showing the potential implications of the social beliefs.

Before presenting the regression estimates, we established that the types of vignettes were randomly presented to the respondents. We found no substantial differences among the respondents who were presented with the different combinations of the vignettes based on socioeconomic characteristics, prevalence of toilet ownership, and social expectations and beliefs (Table 4). For instance, the average number of males and females across all the groups was similar. However, we found statistically significant differences for certain groups for some of the sociodemographic variables. For example, a statistically significant difference of 4% was found between Muslim respondents who were presented with a low EE–low NE vignette and those assigned a high EE–high NE vignette. A similar difference was observed among socially advantaged group (other category) respondents being assigned with low EE–low NE and high EE–low NE vignettes. Nevertheless, it can be argued that even after randomization, there can be a slight possibility of statistically significant differences among some variables.^34^ We accounted for the differences in our estimation strategies using control variables in order to make causal estimates.

**Table 4:** Comparison of basic variables across different combinations of vignettes

Variable	Low EE–Low NE	High EE–Low NE	Low EE–High NE	High EE–High NE	Difference					
	(1)	(2)	(3)	(4)	(1)–(2)	(1)–(3)	(1)–(4)	(2)–(3)	(2)–(4)	(3)–(4)
Public toilet usage	0.63	0.63	0.62	0.64	0.002	0.01	−0.01	0.01	−0.02	−0.03
Actual EE	5.25	5.13	5.34	5.51	0.12	−0.08	−0.26	−0.2	−0.37	−0.17
Actual NE	4.4	4.62	4.51	4.64	−0.23	−0.11	−0.25*	0.12	−0.02	−0.14
Gender										
Male	0.49	0.49	0.51	0.51	0.001	−0.02	−0.02	−0.02	−0.03	−0.002
Female	0.51	0.51	0.49	0.49	−0.001	0.02	0.02	0.02	0.03	0.002
Religion										
Hindu	0.81	0.78	0.8	0.78	**0.03** *	0.009	**0.03** *	−0.02	0.001	0.02
Muslim	0.14	0.17	0.17	0.18	−**0.03**†	−0.02	−**0.04**‡	0.006	−0.01	−0.02
Other	0.05	0.04	0.03	0.04	0.001	0.01	0.01	0.01	0.009	−0.001
Social group										
SC/ST	0.41	0.4	0.39	0.42	0.02	0.02	−0.001	0.008	−0.02	−0.03
Other	0.2	0.24	0.22	0.21	−**0.04**†	−0.02	−0.01	0.02	**0.02** *	0.008
OBC	0.33	0.31	0.33	0.32	0.02	−0.002	0.01	−0.02	−0.005	0.02
Education										
No education	0.31	0.33	0.31	0.32	−0.03	−0.005	−0.02	0.02	0.009	−0.01
Primary	0.13	0.12	0.13	0.13	0.006	−0.001	−0.005	−0.007	−0.01	−0.003
Secondary	0.32	0.31	0.33	0.3	0.01	−0.009	0.02	−0.02	0.008	0.02
Higher	0.24	0.23	0.23	0.24	0.008	0.02	0.001	0.007	−0.006	−0.01
Assets										
0	0.26	0.27	0.28	0.27	−0.01	−0.02	−0.004	−0.005	0.006	0.01
1	0.32	0.31	0.32	0.31	0.004	−0.006	0.004	−0.01	−0.0003	0.01
2	0.26	0.24	0.24	0.25	0.01	0.02	0.004	0.003	−0.009	−0.01
3	0.16	0.17	0.16	0.17	−0.009	0.002	−0.005	0.01	0.004	−0.008

EE = empirical expectation; NE = normative expectation; OBC = other backward class; SC/ST: scheduled caste/scheduled tribe. This table reports the *t* test results for the group of basic variables across the different combinations of the vignette. It shows that the subset of vignettes sent to different respondents across various groups are similar. The study uses the LENNS Survey data from Bihar and Tamil Nadu, 2018.^33^ Bold indicates values that are statistically significant.

* *P* < 0.1 level.

† *P* < 0.05 level.

‡ *P* < 0.01 level.

**Figure 2.**
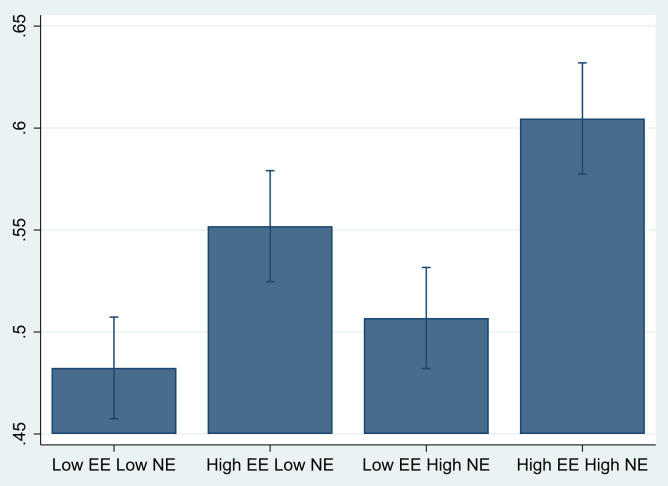
Regression estimates from randomly administered vignettes. The predicted probabilities from the probit regression model using “margins” command in STATA 15 are presented with 95% CIs calculated by clustering the standard errors at the PSU levels. EE = empirical expectation; NE = normative expectation; PSU = primary sampling unit.

Figure 2 represents the predictive probability calculated from the main regression equations with the vignettes. As seen here, when respondents were presented with the hypothetical low EE–low NE vignette, 48% projected that the hypothetical vignette character would use public toilets alone when living in a community. In a setting with low EE and high NE, almost half (51%) of respondents projected that a woman would use public toilets alone. We noted the highest potential impact when respondents were exposed to high EEs. The probability of women using public toilets alone increased to 55% for the high EE and low NE and 60% for the high EE and high NE social beliefs. These estimates remained similar for urban, semi-urban, and rural areas (see Supplemental Figure 1). We tested whether differences in the marginal effects across urban, semi-urban, and rural areas were similar and found them to be statistically insignificant. The findings also remained largely consistent across Bihar and Tamil Nadu (Supplemental Figure 2). Overall, the results underscore the importance of EEs in influencing behavior, which corroborates with other studies in the context of toilet usage and exclusive breastfeeding.^31^^,^^32^ Our findings suggest that the inferences drawn from the data are consistent across all three settings examined in our study.

## DISCUSSION

Our study results indicate that the perceived prevalence of women within the community going outside alone to use public toilets influenced PNBs about whether it is right for young women to go out alone to use public toilets. These beliefs were also positively associated with a higher level of perceived approval for women in the community to use public toilets alone. Findings from the hypothetical vignette analysis suggest that women are more likely to leave home alone to use public toilets in settings where one believes that many women in their communities also use them alone.

This paper contributes to multiple strands of the literature. Our findings inform the problem of non-usage of public toilets, which is a significant concern for both public health and gender equality.^5^^–^^7^ Second, we have also contributed to the growing literature on issues related to gender norms, which restrict women from using public toilets.^13^^,^^14^ The findings from our study will also be relevant to other low- and middle-income countries with issues of public toilet usage among women because of existing gender norms, which restricts their mobility. Lastly, we provide an alternative way to elicit a potential causal link between behavior and social expectations. The methodology used in our study is useful in obtaining the causal impacts of a behavior conditional on social expectations, especially in a context such as public toilet usage, where conducting randomized control trials may not be feasible. We use the findings from our study to inform policy and behavioral interventions to shift social expectations in order to see a change in the individual. Multiple interventions have been conducted in the past to improve public toilet usage across South Asian countries. However, most of these interventions tried to alter the behavior without focusing on the sociocultural aspects around the non-usage of public toilets.^35^^,^^36^ Our study tries to bridge this gap and contributes toward altering the social norms and developing potential interventions to improve public toilet usage among women. Our results show the importance of perceptions of common behavior as a critical determinant of one’s own perception of public toilet usage. Previous studies recommended behavioral interventions to improve public toilet usage by women^37^^–^^39^; however, none have focused on the social expectations and beliefs that these interventions may include. The results from our study highlight that projecting what others around us do and approve of are powerful drivers to shift personal beliefs and to encourage independent usage of public toilets among women.

Restrictive gender norms affect females’ mobility and thus limit their ability to take advantage of public toilets,^40^^,^^41^ further deteriorating their health and well-being.^8^^,^^42^ Shifting these norms might be challenging and necessitates the engagement of relevant community members to promote adoption of a previously prohibited behavior, as improved sanitation facilities make it easier for women to access public toilet facilities. A core group of motivated community members are required to raise collective and individual awareness of public toilet usage by women. Then, as these enthusiastic members reach out to other community members, their conception of public toilet usage could be different, which could perhaps assist in the convergence of motivations. Unease among relevant members can be explored throughout discussions that produce new aspirations for women in one’s community. Once this is done, the improvement in public toilet usage by women alone will serve as a signal to facilitate a collective behavioral change in public toilet usage by women.

Despite multiple liberal policies and practices, including the Swachh Bharat Mission, restrictive gender norms limit the ability of women to use public toilets. Hence, innovative implementation strategies are required to target restrictive gender norms. Growing actions to bring in social change, which are linked in creative ways via social media, hold promise to enhance equitable access and services for women to use public toilets themselves.^43^ These social movements are required to bring collective action to the forefront, given the prevalence of gender inequity and restrictive gender norms.

Government, policymakers, workers from civil society organizations, and other specialists should reshape social beliefs to change personal beliefs. Following the Swachh Bharat Mission, many people have started building toilets or using nearby public toilets. One of the best strategies in this scenario could be targeting the social beliefs of these individuals to generate social pressure on the public to accept women using public toilets alone.

Social norm messaging is one of the most common strategies policymakers adopt to change social norms. It has been successfully employed in various context in shifting such as voter turnouts, sustainable transportation, and handwashing.^44^^–^^46^ If community members broadcast information that many women are accessing public toilets and that it is acceptable to do so, it will project to the target audience that it is safe and acceptable to do so. Making it common for young women to feel comfortable using public toilets alone in their community will influence others like them to do the same. In time, it can lead to the emergence of a new norm whereby public toilets are perceived to be safe.^47^

Although structural improvements and maintenance of public toilets are necessary, engaging community norms is key in boosting their use. Community-wide messaging can be done by (1) local audience, who have tied up with the health and local NGOs, (2) household heads within their families (3) by using the power of social networks and spreading the message. Community-wide messaging helps bring behavioral change as the number of people adopting the target behavior increases, and it can empower women to use public toilets alone. However, we also need to take structural issues around public toilets into account. Two major factors surrounding public toilet usage for women are the poor infrastructure and lack of safety. To improve public toilet usage among women, the necessary precursor is to fix the structural issues around it. The behavioral interventions may lead women to use the public toilets; however, usage can only be sustained if the surrounding structural issues are resolved.

## LIMITATIONS

There are certain limitations in this paper. India is a diverse country with different cultures coming together across various geographies. We tried to capture these differences via the data from the two diverse states (i.e., Bihar and Tamil Nadu). However, generalizing data to a local context that differs from these two states might be inappropriate. In this study, we did not include data from individuals who did not live in slum areas or to those who live in unregistered slums. These will be important considerations if these findings are extended into settings with varying levels of access to public sanitation facilities and different sociodemographic settings.

Next, our analysis using vignettes helped us predict the likelihood of perceptions of public toilet usage by women alone. The situation was presented verbally as a thought exercise. Although we tested the framing of the item, it is possible that some respondents focused on social beliefs of young women “*going alone*” instead of “*using public toilets alone.*” Our estimates did not allow us to estimate actual changes in behavior due to real-life changes in social beliefs. Future researchers can create interventions that randomly manipulate the social beliefs around public toilet usage by women alone and see its effect on behavior around actual public toilet usage by women alone.

## CONCLUSIONS

This paper examined how PNBs surrounding toilet usage by women alone were related to social expectations. Our results provide further evidence that perceived beliefs of what women in one’s community do or approve of do influence personal beliefs. Estimations using experimentally manipulated vignettes also indicate that women are more likely to use public toilets alone if they live in a community where other women also use them alone. Policymakers should consider social beliefs about what others do to shift gender norms and improve public space access and service utilization. These findings can help policymakers, local NGOs, and other stakeholders create interventions to bring about behavioral change contingent upon social beliefs.

## Supplemental Materials


Supplemental materials

## Footnotes

† This variable was reverse coded to keep the directionality the same for both empirical and normative expectations.

‡ State FE was applied only in column (4) and is not applicable for column (5)-(6).

§ These results might be driven by low sample size (102).

‖ Our results did not change even when we used logistic regression instead of probit (Supplemental Table 3).
