# Supplementary material for: Unlocking the Role of Social Norms: How They Shape Women’s Public Toilet Usage in India
Source: Am J Trop Med Hyg. 2023 Oct 9;109(5):1177–86. doi: 10.4269/ajtmh.23-0220 (PMC10622457; doi:10.4269/ajtmh.23-0220)
Supplement: Supplementary file 1 [file tpmd230220.SD1.pdf]

APPENDIX

Table A1. Variable Definitions

Variable	Description
Outcome Variable	
Individual Beliefs	Society may think it is right or wrong for a woman between the ages of 16 and 30 to leave the home alone to use the public toilet. do you personally think it is right, neither right nor wrong, or wrong for a woman between the ages of 16 and 30 to leave the home alone to use the public toilet?<br><br>Options: right, neither right or wrong, wrong.<br><br>Our outcome variable takes the value 1 if the respondents answer right; 0 otherwise.
Vignette	Please imagine an area similar to where you live.<br><br>A young woman from your area, whom you don't know, moved there one year ago.<br><br>She uses a public toilet when she needs to defecate.<br><br>She learned that [most]/[few] people disapprove of women going to the public toilet alone [and]/[, but] she also learned that [most/few] women go alone.<br><br>What do you think she will do?

	Four different categories of vignettes were created i.e., low EE low NE to high EE and high NE.<br><br>lowEE lowNE denotes the type of vignette that has low EE (few women go alone to use public toilets) as well as low NE (most people disapprove of women going to the public toilet alone). Similarly, lowEE highNE denotes that type of vignette, which has low EE (few women go alone to use public toilets) but high NE (few people disapprove of women going to the public toilet alone). The others follow the same rule.<br><br>The vignette was followed by three response options i.e., Won't go alone to the public toilet, don't know/equally likely, and will go alone to the public toilet.<br><br>Our outcome variable takes the value 1 if the respondents answer will go alone to the public toilet; 0 otherwise.
Interest Variables	
Empirical Expectations	Out of ten women between the ages of 16 and 30 in your community, the last time they left the house to use the public toilet, how many do you think did so alone?<br><br>This a continuous variable with values ranging from 0 to 10

Normative Belief	Out of ten members in your community, how many do you think believe that it is right for a woman between the ages of 16 and 30 to leave the home alone to use the public toilet?<br><br>This a continuous variable with values ranging from 0 to 10
Other Controls	
Gender	1 for females and 0 for males. Reference Group: Male
Social Group	1 for Other, 2 for Other Backward Class and 3 for Scheduled Class/ Scheduled Tribe. Reference Group: SC/ST.
Toilet	1 for respondents who have private toilets or share between households; 0 otherwise; Reference: Respondents with no private toilet.
Education Level	1= No Education; 2=Up to Grade 5; 3= Up to Grade 10; 4= 11th and above. Reference Group: No Education.
Assets	3 for respondents having motorcycle, fridge and color television; 2 for respondents having any two among motorcycle, fridge and color television; 1 for respondents having one among motorcycle, fridge and color television. Reference Group: 0: for respondents without motorcycle, fridge and color television.
PSU Type	1 for Municipal Corporation, 2 for Town Panchayat and 3 for Gram Panchayat. Reference Group: Municipal Corporation.

Table A2. Marginal effects to estimate the association of empirical and normative expectations on the personal normative beliefs of public toilet usage by women alone across different PSU.

	Municipal Corporation	Town Panchayat	Gram Panchaya t
Leaving house alone to use public toilets (EE)	0.04***	0.03**	0.02
	(0.005)	(0.008)	(0.015)
Approval of leaving the house alone to use public toilets (NE)	0.06***	0.06***	0.03**
	(0.004)	(0.004)	(0.012)
Gender			
Male			
Female	0.09*	0.01	-0.01
	(0.048)	(0.049)	(0.113)
Social Group			
Other			
OBC	0.03	0.14	0.41***
	(0.069)	(0.094)	(0.055)
SC/ST	-0.01	0.13**	0.26***

	(0.053)	(0.059)	(0.070)
Owns a toilet			
No			
Yes	0.01	-0.02	-0.01
	(0.033)	(0.058)	(0.042)
Education			
No Education			
Primary	0.06	-0.04	-0.11
	(0.060)	(0.052)	(0.149)
Secondary	-0.01	-0.03	-0.01
	(0.036)	(0.040)	(0.084)
Higher	0.06	0.01	-0.31**
	(0.052)	(0.065)	(0.133)
Assets			
0			
1	-0.03	0.01	-1.14***
	(0.050)	(0.077)	(0.071)
2	-0.03	-0.09	-1.00***
	(0.049)	(0.071)	(0.089)
3	-0.09	0.02	-1.01***

	(0.056)	(0.070)	(0.138)
District FE	Y	Y	Y
State FE	Y	Y	Y
PSU Type FE	Y	Y	Y
PSU Name FE	Y	Y	Y
Pseudo R2	0.35	0.44	0.30
Observations	728	428	102

The dependent variable is whether it is right or wrong for a woman between the ages of 16 and 30 to leave the home alone to use the public toilet. *** p < 0.01, ** p < 0.05, * p < 0.1 level. EE stands for Empirical Expectations and NE stands for Normative Expectations. Table A2 gives the marginal effects from the probit regression outlined in equation (1), which reports the results of social beliefs (EE and NE) on PNB. We present the indicators of EE and NE together, along with the controls across the PSUs. These three columns have the most comprehensive set of controls that allow us to get close to unbiased estimates and include District, State, PSU Type, and PSU fixed effects. All the standard errors were clustered at PSU levels.

Table A3. Marginal effects to estimate the association of empirical and normative expectations on the personal normative beliefs of public toilet usage by women alone using logistic regression.

	It is right for women to use public toilets alone (PNB)					
	Full sample				Bihar	Tamil Nadu
VARIABLES	(1)	(2)	(3)	(4)	(5)	(6)
Leaving house alone to use public toilets (EE)	0.05***		0.03***	0.03***	0.02**	0.04***
	(0.003)		(0.003)	(0.005)	(0.008)	(0.004)
Approval of leaving the house alone to use public toilets (NE)		0.07***	0.06***	0.06***	0.06***	0.06***
		(0.001)	(0.002)	(0.003)	(0.005)	(0.003)
Gender						
Male						
Female				0.06	-0.05	0.12***
				(0.038)	(0.048)	(0.025)
Social Group						
SC/ST						
Other				0.10	-0.02	0.12**
				(0.057)	(0.111)	(0.039)
OBC				0.04	-0.02	0.07**
				(0.043)	(0.057)	(0.033)
Owns a toilet						

No						
Yes				-0.02	-0.01	-0.03
				(0.031)	(0.054)	(0.029)
Education						
No Education						
Primary				0.02	0.13*	-0.04
				(0.042)	(0.070)	(0.041)
Secondary				-0.02	-0.08	-0.04
				(0.028)	(0.070)	(0.035)
Higher				0.02	0.13*	-0.05
				(0.043)	(0.075)	(0.037)
Assets						
0						
1				-0.01	-0.03	0.09
				(0.039)	(0.066)	(0.062)
2				-0.02	-0.12	0.09
				(0.038)	(0.077)	(0.064)
3				-0.02	-0.14	0.11
				(0.040)	(0.085)	(0.067)
District FE	N	N	N	Y	Y	Y

State FE	N	N	N	Y	Y	Y
PSU Type FE	N	N	N	Y	Y	Y
PSU Name FE	N	N	N	Y	Y	Y
Pseudo R2	0.09	0.25	0.27	0.36	0.34	0.4
Observations	1345	5052	1345	1258	308	950

The dependent variable is whether it is right or wrong for a woman between the ages of 16 and 30 to leave the home alone to use the public toilet. *** $p < 0.01$, ** $p < 0.05$, * $p < 0.1$ level. EE stands for Empirical Expectations and NE stands for Normative Expectations. Table 4 gives the marginal effects from the probit regression outlined in equation (1), which reports the results of social beliefs (EE and NE) on PNB. We present six specifications: the first and second specification has the indicators of EE and NE individually in the model without any controls; the third specification has the indicators of EE and NE together; the fourth specification has the indicators of EE and NE together, along with the controls; Fifth and sixth specification is similar to forth specification; however, it is restricted to the data in Bihar and Tamil Nadu, respectively. The last three specifications have the most comprehensive set of controls that allow us to get close to unbiased estimates and include District, State, PSU Type, and PSU fixed effects. All the standard errors were clustered at PSU levels.

SC/ST: Scheduled Caste/Scheduled Tribe; OBC: Other Backward Classes.

Figure F1. Regression estimates from randomly administered vignettes across the PSUs

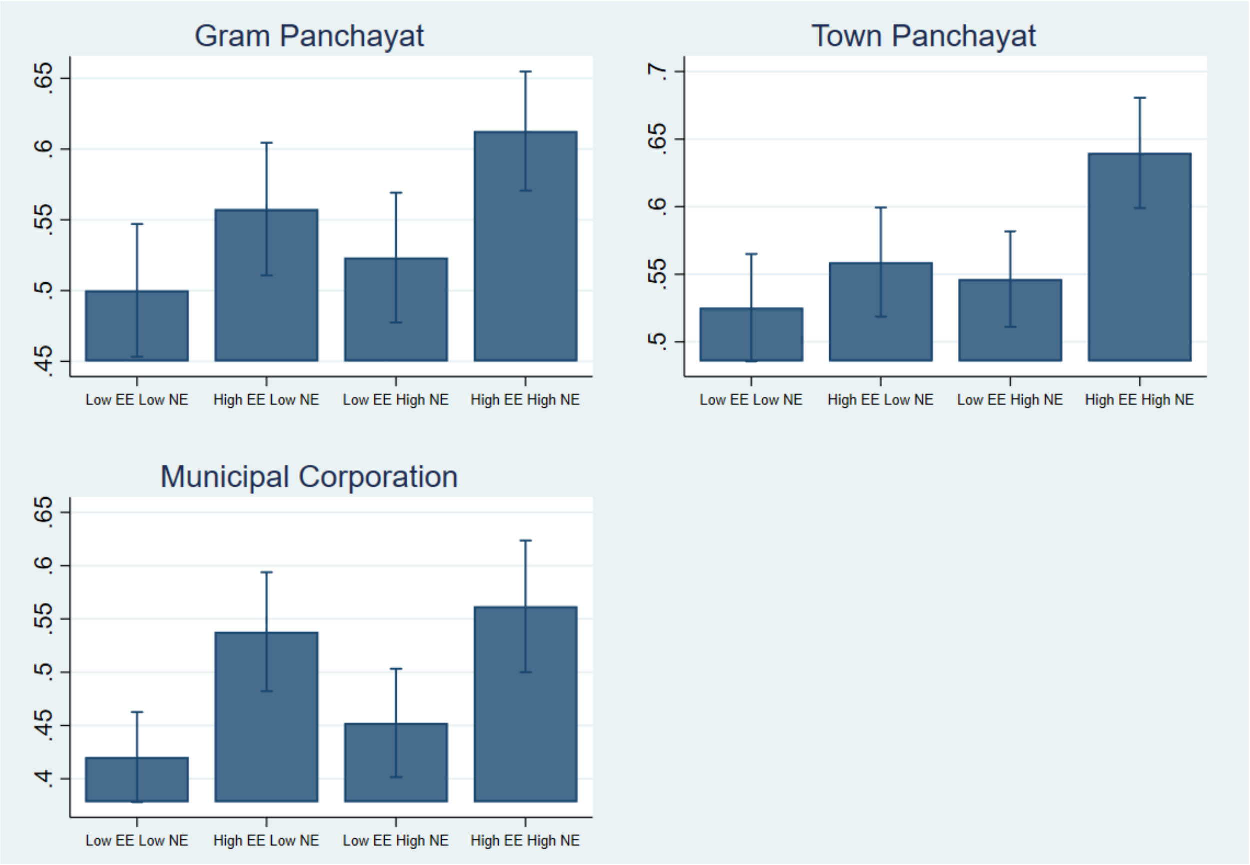

Footnote: The predicted probability from probit regression model using “margins” command in STATA 15 are presented with 95% confidence interval calculated by clustering the standard errors at the PSU levels.

Figure F2. Regression estimates from randomly administered vignettes across the states

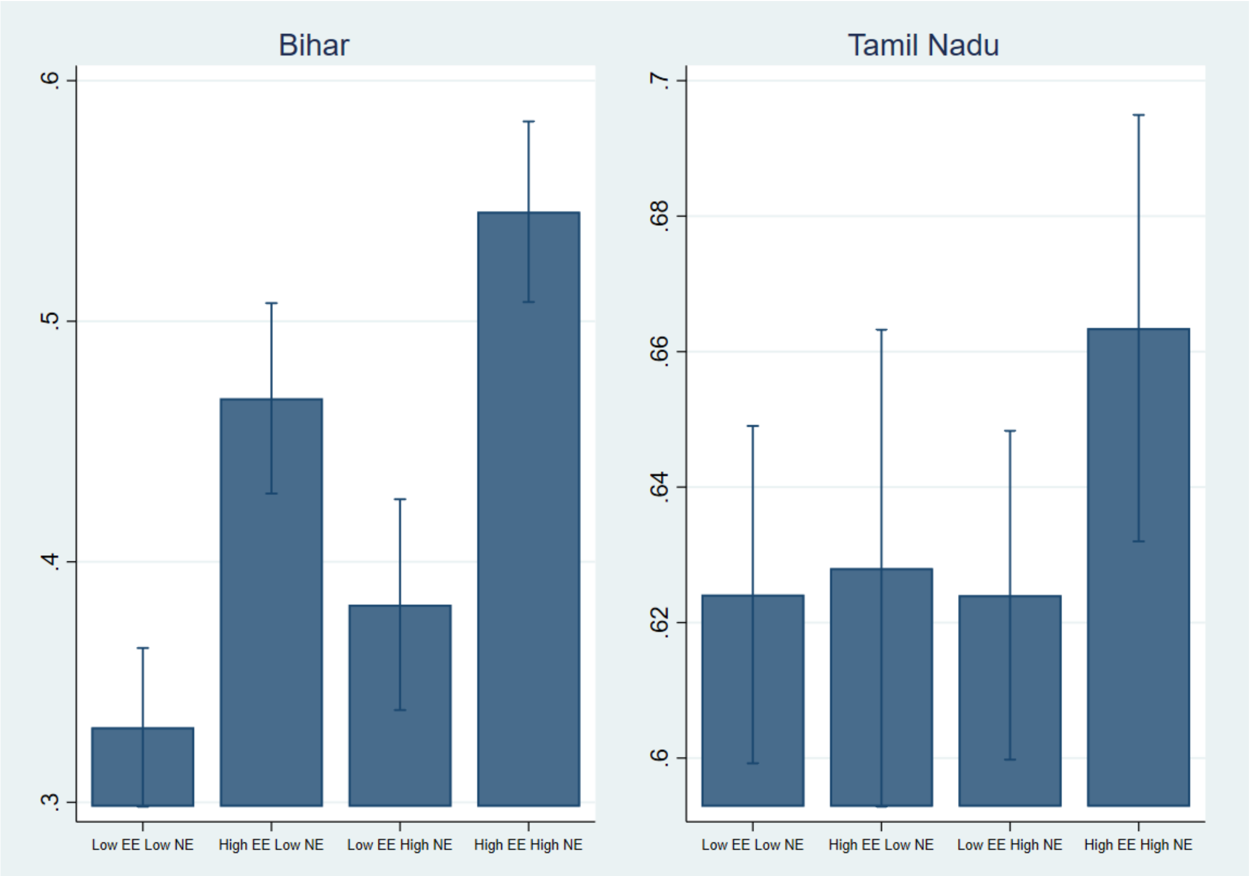

Footnote: The predicted probability from probit regression model using “margins” command in STATA 15 are presented with 95% confidence interval calculated by clustering the standard errors at the PSU levels.
